# L-Selenomethionine Alleviates Cryo-Induced Ferroptosis Through the NRF2–SLC7A11–GPX4 Pathway, Improving Post-Thaw In Vitro Quality of Dairy Goat Spermatozoa

**DOI:** 10.3390/antiox15030392

**Published:** 2026-03-20

**Authors:** Zi-Tao Jiang, Shun-Kai Yang, Xu-Dong Zhou, Xu Zhang, Zi-Tong Hu, Song-Mao Guo, Guo-Yu Zhang, Shuai-Qi Han, Fei Wen, Xiao-Xu Chen, Jian-Hong Hu

**Affiliations:** College of Animal Science and Technology, Northwest A&F University, Yangling 712100, China; jiangzitao19@163.com (Z.-T.J.); ysk248650@nwafu.edu.cn (S.-K.Y.); zhouxudong1999@126.com (X.-D.Z.); zx02170314@126.com (X.Z.); huzitong2024@163.com (Z.-T.H.); 18854883308@163.com (S.-M.G.); zzr425983391@126.com (G.-Y.Z.); hshuaiqi@nwafu.edu.cn (S.-Q.H.); wenfei2023@nwafu.edu.cn (F.W.)

**Keywords:** dairy goat, ferroptosis, sperm cryo-damage, oxidative stress

## Abstract

Background: Cryopreservation induces oxidative stress, membrane disruption, and mitochondrial injury in spermatozoa, leading to impaired motility and fertility. Selenium, as an essential trace element, protects cells from oxidative damage through selenoproteins such as glutathione peroxidase 4 (GPX4), a critical enzyme that detoxifies lipid hydroperoxides and inhibits ferroptosis. This study investigated whether supplementation with L-selenomethionine (L-SeMet), an organic selenium source with superior bioavailability and lower toxicity than inorganic forms, could alleviate cryo-induced sperm injury by suppressing ferroptosis. Methods & Results: Dairy goat sperm were cryopreserved with 0, 2, 4, 6, 8, 10 μM L-SeMet. Supplementation with 6 μM L-SeMet significantly improved motility, membrane and acrosome integrity, and mitochondrial membrane potential. Biochemical assays showed reduced iron, ROS, and MDA levels, alongside increased ATP, SOD, and GSH contents. Proteomic analysis identified 148 differentially expressed proteins, including up-regulation of GPX4, FTH1, VDAC2, and VDAC3—core ferroptosis regulators. Metabolomic profiling further revealed enrichment in unsaturated fatty acid biosynthesis, amino acid metabolism, and the TCA cycle, pathways closely linked to ferroptosis regulation. Transmission electron microscopy confirmed that L-SeMet preserved mitochondrial ultrastructure. Mechanistically, L-SeMet mirrored the ferroptosis inhibitor N-acetyl-L-cysteine and reversed RSL3-induced oxidative damage. Western blotting verified activation of the NRF2–SLC7A11–GPX4 antioxidant axis and inhibition of KEAP1 expression. Conclusions: Collectively, these findings demonstrate that L-SeMet protects spermatozoa from cryo-induced injury by stabilizing redox homeostasis, maintaining mitochondrial function, and inhibiting ferroptosis. The results highlight ferroptosis as a critical mechanism of sperm cryodamage and identify L-SeMet as a promising metabolic intervention to enhance post-thaw sperm quality and fertility.

## 1. Introduction

Cryopreservation underpins assisted reproduction, genetic resource banking, and livestock improvement, yet post-thaw sperm frequently exhibit reduced motility with concomitant membrane and mitochondrial defects attributed to oxidative stress and lipid peroxidation [1,2]. Among regulated cell-death programs, ferroptosis—an iron-dependent, phospholipid-peroxidation–driven process—offers a mechanistic framework linking iron overload, mitochondrial dysfunction, and membrane damage under freeze–thaw stress [3]. Recent syntheses further distinguish ferroptosis from apoptosis/necroptosis and emphasize its preventability by iron chelators or lipid-soluble antioxidants [4,5]. In male reproduction, emerging clinical and experimental data implicate ferroptosis markers and pathways (e.g., NRF2–SLC7A11–GPX4) in spermatogenic injury and infertility phenotypes [6,7].

Selenium-based interventions have been explored to bolster antioxidant capacity, but reported benefits on CASA metrics and membrane stability vary across species, extenders, and selenium chemistry (e.g., selenomethionine vs. nano-selenium), suggesting dose- and matrix-dependence [8]. A biologically plausible route for benefit is selenium provisioning to GPX4 and allied ferroptosis-defense nodes: recent studies show that selenium improves testicular/sperm outcomes by upregulating GPX4 and relieving oxidative/iron stress, while reviews reaffirm GPX4’s central role in safeguarding the sperm midpiece [9,10]. Functionally, mitochondrial membrane potential (MMP) and ATP production are tightly coupled to sperm kinematics, with JC-1–based MMP readouts correlating with motility/progressive movement and with improved methodological standardization for reproductive assessment [11,12]. These concepts are directly relevant to cryo-injury, where mitochondrial dysfunction and oxidative damage co-evolve to depress post-thaw motion performance [13].

Despite these insights, the specific contribution of ferroptosis to sperm cryo-injury and whether L-selenometionine (L-SeMet) can mitigate this process by engaging the NRF2–KEAP1–SLC7A11–GPX4 axis remain incompletely resolved [14,15].

We hypothesize that L-SeMet, within a defined therapeutic window, preserves midpiece bioenergetics and limits iron-dependent lipid peroxidation during freezing–thawing by engaging the NRF2–KEAP1–SLC7A11–GPX4 axis, thereby improving post-thaw motility. To test this hypothesis, we (i) determine the effective L-SeMet dose using computer-assisted sperm analysis (CASA) with assessments of plasma-membrane/acrosome integrity and mitochondrial membrane potential (MMP); (ii) map supplementation-associated alterations with untargeted metabolomics and DIA-based proteomics to identify pathway-level signals; (iii) transmission electron microscopy (TEM) provides ultrastructural corroboration of mitochondrial integrity. Validate pathway nodes and functional redox/iron readouts by immunoblotting and biochemical assays; and (iv) anchor specificity with orthogonal perturbations, comparing L-SeMet with a glutathione augmenter N-acetylcysteine (NAC) and challenging with an RSL3, both with documented relevance to redox/ferroptosis biology in reproductive or tissue models [16].

Conceptually, this work positions ferroptosis control as a tractable target for sperm cryopreservation and clarifies how selenium biology intersects with midpiece energetics and membrane stability. Practically, establishing a dose window for L-SeMet could inform standardized extender formulation across ejaculates and breeds, while the multi-layer readout set (CASA, mitochondrial function, omics, pathway proteins, and iron/oxidative indices) offers a deployable framework to screen cryoprotective additives and to connect molecular rescue with fertilization and embryo development outcomes [1,2].

## 2. Materials and Methods

### 2.1. Semen Collection and Cryopreservation

During the breeding season from mid-August to early November 2024, semen was collected twice weekly from 5 healthy 2-year-old Guanzhong dairy goats at Aonik (Shaanxi Fuping) using an artificial vagina. Immediately after collection, sperm motility was assessed using the CASA system (IVOS II, IMV, L’Aigle, France), and sperm concentration was measured with a sperm densitometer. Semen samples from 5 dairy goats that met the criteria of total motility exceeding 80% and a concentration of 2–3 × 10^9^ cells/mL on the same day were selected for the experiment. Each buck served as an independent experimental unit, and semen from each individual was processed, analyzed, and statistically evaluated separately for each treatment. The semen was maintained at 37 °C and diluted to a concentration of 2 × 10^8^ cells/mL with extender I. Seminal plasma was not removed prior to dilution; ejaculates were processed without any centrifugation/washing step.

The base extender I was prepared by dissolving 4.6 g of lactose, 3.1 g of glucose, and 1.5 g of citric acid in ultrapure water, to which 5 mL of penicillin-streptomycin solution (15140122, Gibco, Grand Island, NY, USA) and 15 mL of egg yolk were added, and the volume was adjusted to 100 mL with ultrapure water. Extender II was prepared by supplementing base extender I with glycerol to a final concentration of 6% (*v*/*v*), with gentle mixing until homogeneous. In experiment I, L-selenomethionine (L-SeMet; CAS 3211-76-5; MCE, Monmouth Junction, NJ, USA) was dissolved in sterile ultrapure water to prepare a 10 mM stock solution, aliquoted, and stored at −20 °C. On the day of freezing, a 1 mM working solution was prepared from the stock and added to extender I to achieve final concentrations of 2.0, 4.0, 6.0, 8.0, and 10 µM. This dose–response design was used to evaluate the effects of L-SeMet supplementation on semen cryopreservation outcomes. In experiment II, following dilution with extender I, the semen samples were divided into three groups: one group was supplemented with 5 μM NAC, another with 6 μM L-SeMet, and the third served as a control with no additives. In experiment III, semen samples were allocated into four groups: a control group with no additives; a ferroptosis-inducing group exposed to 5 µM RSL3 in the extender I for 1 h; a group supplemented with 6 μM L-SeMet; and a group treated with both 5µM RSL3 (exposed for 1 h) and 6 μM L-SeMet.

The semen samples were first diluted with Extender I and equilibrated at 4 °C for 2 h. The samples were then adjusted to 1 × 10^9^ cells/mL. Subsequently, the diluted semen was mixed with Extender II at a 1:2 (*v*/*v*) ratio and equilibrated at 4 °C for an additional 1.5 h, resulting in a final glycerol concentration of 4% (*v*/*v*) prior to freezing. The diluted semen samples were loaded into 0.25-mL straws (IMV Technologies, L’Aigle, France) and sealed. Using a programmable freezing device (Cryologic Ltd., Blackburn, VIC, Australia), the straws were cooled to −5 °C at a rate of 1 °C/min. Once the temperature reached −5 °C, the straws were placed 3 cm above the surface of liquid nitrogen for 8 min. Thereafter, the straws were directly plunged into liquid nitrogen for storage and subsequent experimental use [17].

### 2.2. Analysis of Sperm Quality by CASA System

For each evaluation, three straws were thawed in a water bath at 37 °C (30 s), and the semen samples were pooled. A 10 μL volume of the semen sample was placed on a pre-warmed (37 °C) glass slide (Minitube, Tiefenbach, Germany). Sperm motility and other motility parameters were assessed by a specialist using a computer-assisted sperm analysis (CASA) system (HVIEW-SSA V8.0, Fuzhou Hong Vision Software Technology Co., Fuzhou, China) at a magnification of 400× across five distinct fields within the same sample. All experiments, including semen freezing, were replicated five times.

### 2.3. Assessment of Sperm Acrosome Integrity and Plasma Membrane Integrity

Sperm membrane integrity was analyzed using the double staining kit of SYBR-14/propidium iodide (PI) (L7011, Thermo Fisher Scientific, Waltham, MA, USA) [18,19]. Semen samples were placed in 1.5 mL centrifuge tubes, and SYBR-14 staining solution was added and incubated for 10 min. The samples were then incubated with PI staining solution for an additional 10 min. Finally, 10 µL of the stained semen sample was placed on a glass slide and immediately observed under a Leica DM6 B fluorescence microscope (Leica Microsystem, Wetzlar, Germany). Each experiment was replicated five times, and all procedures were conducted in a dark room to preserve the stability of the fluorescent dyes.

The acrosomal integrity of cryopreserved dairy goat spermatozoa was assessed using fluorescein isothiocyanate-conjugated peanut agglutinin (FITC-PNA) staining. Following thawing, the pooled sperm samples were washed three times with PBS. Subsequently, 30 µL of the sperm suspension was placed on a glass slide and fixed with absolute methanol for 10 min. FITC-PNA (10 µg/mL) was then applied to the fixed samples and incubated at 37 °C for 30 min. After incubation, the slides were stained with DAPI (1 µmol/L) in the dark at room temperature for 10 min. The slides were examined using a Leica DM6 B fluorescence microscope (Leica Microsystems, Germany) equipped with a 40× objective (total magnification, 400×) to evaluate acrosomal integrity [20] (at least 100 sperm per field; five fields per sample).

### 2.4. Detection of MMP

The mitochondrial membrane potential (MMP) of post-thawed sperm was measured using the MMP assay kit (M8650, Solarbio, Beijing, China) [21]. JC-1 working solution was prepared by diluting JC-1 (200×) at 50 μL per 8 mL ultrapure water, followed by addition of 2 mL 5× staining buffer (final 1× working solution), according to the manufacturer’s instructions. Sperm from different groups were mixed with an equal volume of JC-1 working solution and incubated at 37 °C in the dark for 20 min. After incubation, the samples were centrifuged at 300 rpm, resuspended in PBS, and washed three times. Fluorescence intensity was measured using a microplate reader (BIOTEK, Winooski, VT, USA) at an excitation wavelength of 525 nm and an emission wavelength of 590 nm. Additionally, 30 μL of diluted semen from each group was spread evenly on a glass slide and air-dried. The slides were examined using a Leica DM6 B fluorescence microscope (Leica Microsystems, Germany) equipped with a 40× objective (total magnification, 400×). According to the manufacturer’s instructions, sperm with red fluorescence were identified as having high MMP (JC-1 aggregates), while those with green fluorescence were considered to have low MMP. Each group included at least three biological replicates.

### 2.5. Non-Targeted Metabolomics Plus Analysis

Non-targeted metabolomics was utilized to profile the metabolites within the samples. Post-thawing at 37.5 °C, a portion of each sample was combined with a chilled methanol/acetonitrile/water mixture (2:2:1, *v*/*v*/*v*). This mixture was vortexed and then sonicated at low temperature for 30 min, followed by a 10 min static period at −20 °C. The mixture was subsequently centrifuged at 14,000× *g* for 20 min at 4 °C, with the supernatant being collected and vacuum-dried. For mass spectrometry, the dried samples were reconstituted in 100 μL of an acetonitrile/water solution (1:1, *v*/*v*), vortexed, and centrifuged at 14,000× *g* for 15 min at 4 °C. The resulting supernatant was injected for analysis. The mobile phase for chromatography consisted of A (water with 0.1% formic acid) and B (acetonitrile), delivered at a flow rate of 0.35 mL/min, with an injection volume of 3 μL. Electrospray ionization (ESI) was employed as the ionization source, and mass spectrometry data were collected in both positive and negative ion modes. Separation of the samples was achieved using an Agilent 1290 Infinity LC UHPLC system (Agilent Technologies, Santa Clara, CA, USA) equipped with a HILIC column, maintained at 25 °C, with a flow rate of 0.5 mL/min and an injection volume of 2 μL. The mobile phase for this separation was composed of A (water + 25 mM ammonium acetate + 25 mM ammonia) and B (acetonitrile), also at a flow rate of 0.5 mL/min. Following separation via the Vanquish LC UHPLC system (Thermo Fisher Scientific, Waltham, MA, USA), the samples were subjected to mass spectrometry using an Orbitrap Exploris™ 480 mass spectrometer (Thermo Fisher Scientific, Waltham, MA, USA), with ESI in both positive and negative ion modes.

### 2.6. DIA Quantitative Proteomics Analysis

Samples were thawed at 37.5 °C for 2 min and mixed with SDC buffer (5% sodium lauroyl sarcosinate, 100 mM Tris-HCl, pH 8.5). Dithiothreitol (DTT) was added to reduce disulfide bonds, followed by incubation at 37 °C for 1.5 h. Iodoacetamide (IAA) was then added to alkylate the reduced cysteine residues, and the mixture was reacted in the dark at room temperature for 30 min. Trypsin was added at a weight ratio of 1:50 and incubated at 37 °C for 15–18 h (overnight). The peptides were desalted using an MCX column (Cat. No. OS-MCX-1ML, Omicsolution, Shanghai, China), concentrated by vacuum centrifugation, and reconstituted in 20 μL of water containing 0.1% formic acid. Peptide concentration was estimated by UV absorbance at 280 nm. For DIA experiments, iRT calibration peptides were added. Peptides were analyzed in DIA mode using an Orbitrap™ Astral™ mass spectrometer coupled with a Vanquish Neo UHPLC system (Thermo Fisher Scientific, Waltham, MA, USA). Precursor ions were scanned in the *m*/*z* range of 380–980 with an MS1 resolution of 240,000 at *m*/*z* 200, an AGC target of 500%, and a maximum IT of 5 ms. MS2 scans were performed with 299 windows, an isolation window of 2 *m*/*z*, and an HCD collision energy of 25 eV, with an AGC target of 500% and a maximum IT of 3 ms. The DIA data were analyzed using DIA-NN 1.8.1 software.

### 2.7. Assessment of Iron

The ferrous iron (Fe^2+^) content was detected by using an iron assay kit (E1042, Applygen, Beijing, China) according to the manufacturer’s instructions. Frozen semen straws were thawed at 37 °C for 30 s in a water bath and subsequently washed with PBS. An equal volume of lysis buffer was added to the samples, which were then sonicated for 5 min using an ultrasonic processor. The protein concentrations were quantified through the BCA assay method, employing a BCA assay kit (Product No. PA115, TAINGEN Biotech, Beijing, China) [21]. For the determination of Fe^2+^ levels, 100 μL of a mixture of buffer and 4.5% potassium permanganate solution (1:1) was added to the lysed sample and incubated at 60 °C for 1 h. Subsequently, 30 μL of iron detection reagent was added to the mixture, which was then incubated at room temperature for 30 min. After centrifugation at 12,000 rpm for 5 min, 200 μL of the supernatant was transferred to a clear 96-well plate, and the absorbance at 550 nm was measured using a microplate reader (Synergy H1; BioTek Instruments, Inc., Winooski, VT, USA) to quantify Fe^2+^ levels in the sperm.

### 2.8. Total ROS (tROS) Assay

An ROS assay kit (CA1410, Solarbio, China) was used to measure the ROS level of the post-thawed sperms. The samples were lysed to obtain their protein solutions. Intracellular reactive oxygen species (ROS) can oxidize the non-fluorescent probe DCFH to produce the fluorescent compound DCF. To assess ROS levels, different groups were incubated with a diluted DCFH-DA probe (1:1000, *v*/*v*) in PBS at 37 °C for 20 min in the dark. Fluorescence intensity was measured using a microplate reader (BIOTEK, USA) with excitation and emission wavelengths set at 488 nm and 525 nm.

### 2.9. Evaluation of Oxidative Damage

#### 2.9.1. Malondialdehyde (MDA) Content Assay

Malondialdehyde (MDA) levels were measured using an MDA assay kit (BC0025, Solarbio, China) to evaluate lipid peroxidation of the post-thawed sperms. the protein concentrations were quantified through the BCA assay method, employing a BCA assay kit (PA115, TAINGEN, China). The samples were lysed to obtain their protein solutions. MDA can react with thiobarbituric acid (TBA) under acidic, forming a product that exhibits a maximum absorption wavelength at 532 nm. The concentration of MDA in the samples can be estimated by measuring the absorbance at 532 nm and 600 nm using a microplate reader (BIOTEK, USA).

#### 2.9.2. Assessment of Superoxide Dismutase Activity

SOD activity was assessed using a superoxide dismutase assay kit (BC0175, Solarbio, Beijing, China). Frozen–thawed semen samples were centrifuged, resuspended in extraction buffer to a final concentration of 1 × 10^7^ spermatozoa/mL, and ultrasonically disrupted in an ice bath. After centrifugation, the supernatant was collected, reacted with the working solution according to the manufacturer’s instructions, and the absorbance at 450 nm was measured using a microplate reader (Synergy H1, BioTek Instruments, Winooski, VT, USA).

### 2.10. Measurement of ATP Content

ATP levels in each group were measured using an ATP assay kit (S0026, Beyotime, Shanghai, China). An appropriate volume of ATP assay reagent was diluted with the ATP assay extender at a ratio of 1:9 to prepare the ATP working solution. After thawing and washing, cells from each group were lysed by adding 200 µL of lysis buffer per sample. The lysed samples were then centrifuged at 12,000× *g* for 5 min at 4 °C, and the supernatant was collected. To measure ATP levels, 100 µL of the ATP working solution was mixed with 20 µL of the cell lysate, and the relative luminescence units (RLU) were determined using a microplate reader (BIOTEK, USA).

### 2.11. Assessment of GSH Levels

The concentration of glutathione GSH concentration in different groups was determined using a Reduced Glutathione Content Assay Kit (BC1175, Solarbio, China). The frozen semen samples were thawed in a water bath, washed, and lysed with a cell lysis buffer. The supernatant obtained after centrifugation was mixed with the assay working solution and allowed to react at room temperature for 2 min. The absorbance at 412 nm was measured using a microplate reader (BIOTEK, USA) to quantify the GSH content in the samples.

### 2.12. Transmission Electron Microscopy (TEM)

The subcellular structure and morphology of spermatozoa following cryopreservation and thawing were investigated using transmission electron microscopy (TEM; JEM-1400FLASH, JEOL, Tokyo, Japan), with a focus on mitochondrial alterations under different treatment conditions. Fresh spermatozoa, spermatozoa cryopreserved using a conventional cryoextender, and spermatozoa cryopreserved with a cryoextender containing 6 μM L-SeMet were analyzed. After thawing at 37.5 °C for 5 min, the spermatozoa were incubated for an additional 5 min and then centrifuged at 300 rpm for 5 min. The pellets were washed twice with PBS. The samples were fixed in 2.5% glutaraldehyde at room temperature and post-fixed in 1% osmium tetroxide. Dehydration was performed using a graded series of acetone (30%→50%→70%→80%→90%→95%→100%, with three changes of 100% acetone). The dehydrated samples were infiltrated and embedded in Epon-812 resin using the following ratios of dehydrating agent to embedding medium: 3:1, 1:1, and 1:3. Ultrathin sections (60–90 nm) were prepared using an ultramicrotome and mounted on copper grids. The sections were stained with uranyl acetate for 10–15 min, followed by lead citrate staining for 1–2 min at room temperature. Images were captured using a JEM-1400FLASH transmission electron microscope (JEOL, Japan).

### 2.13. Western Blot Analysis

Proteins from various groups were isolated utilizing a RIPA lysis buffer supplemented with PMSF at a volumetric ratio of 100:1. Subsequently, the protein concentrations were quantified through the BCA assay method, employing a BCA assay kit (Product No. PA115, TAINGEN, China). For the separation of denatured proteins, sodium dodecyl sulfate-polyacrylamide gel electrophoresis was performed at ambient temperature for a duration of 1 h, utilizing a 12% FuturePAGE™ gel (Product No. ET15412Gel, ACE Biotechnology, Xiangtan, China). The proteins were transferred to polyvinylidene fluoride (PVDF) membranes in an ice bath for 1 h. The membranes were blocked with 5% skimmed milk at room temperature for 3 h. The membranes were then incubated with the diluted primary antibody at 4 °C overnight.β-tubulin (10094-1-AP, 1:8000), GPX4 (30388-1-AP 1:5000), NRF2 (16396-1-AP, 1:5000), HO-1 (Ag1190, 1:2000), NQO1 (11451-1-AP 1:5000), Ferritin heavy chain (FTH1) (83428-1-RR, 1:5000), SLC7A11 (26864-1-AP, 1:2000), Following this, the membrane underwent three 10 min washes with Tris-buffered saline Tween-20 (TBST), and was subsequently incubated with the secondary antibody (HRP-Goat Anti-rabbit IgG, diluted at 1:5000) at 37 °C for 3 h. The membrane was subsequently washed and treated with enhanced chemiluminescence (ECL) reagent (34095, Thermo Fisher Scientific, Waltham, MA, USA). Following the reaction, the membrane was visualized using a Gel Doc XR System (BioRad, Hercules, CA, USA). Finally, the protein expression levels were quantified using ImageJ software (version 1.54p, National Institutes of Health, Bethesda, MD, USA).

### 2.14. Statistical Analysis

In the present study, each experiment was replicated at least thrice to ensure reliability. Statistical analyses were performed using GraphPad Prism 8 software. One-way analysis of variance (ANOVA) was employed to assess significant main effects, followed by the least significant difference (LSD) test for post hoc comparisons. The F test was conducted, treating replicate cultures as random effects. Data that did not conform to a normal distribution, as determined by the Shapiro–Wilk test, were subjected to logarithmic transformation. Results are expressed as means ± standard error of the mean (SEM). A *p* value of less than 0.05 or 0.01 was considered to indicate statistical significance. Additionally, different letters were used to denote statistically significant differences (*p* < 0.05).

## 3. Results

### 3.1. L-SeMet Enhances the Cryopreservation Efficiency of Dairy Goat Semen

**Figure 1:**
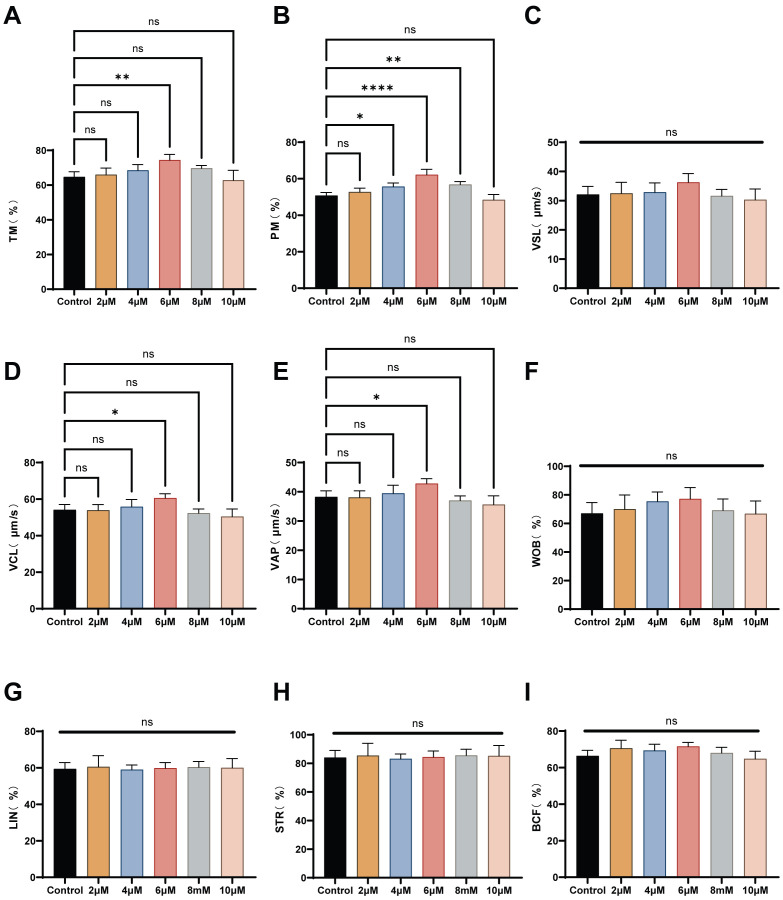
Effects of L-SeMet supplementation on post-thaw sperm motility and kinematic parameters. (**A**–**I**) Quantitative results for progressive motility (PM), total motility (TM), curvilinear velocity (VCL), average path velocity (VAP), straight-line velocity (VSL), amplitude of lateral head displacement (ALH), linearity (LIN), beat cross frequency (BCF), and straightness (STR). Total motility was significantly higher in the 4–8 μM groups compared with the control (*p* < 0.05), and the 6 μM group showed the greatest increase in motility, VCL, and VAP (*p* < 0.05). No significant differences were observed among other parameters (*p* > 0.05). Data are presented as mean ± SEM. ns, not significant; * *p* < 0.05; ** *p* < 0.01; **** *p* < 0.0001.

**Figure 2:**
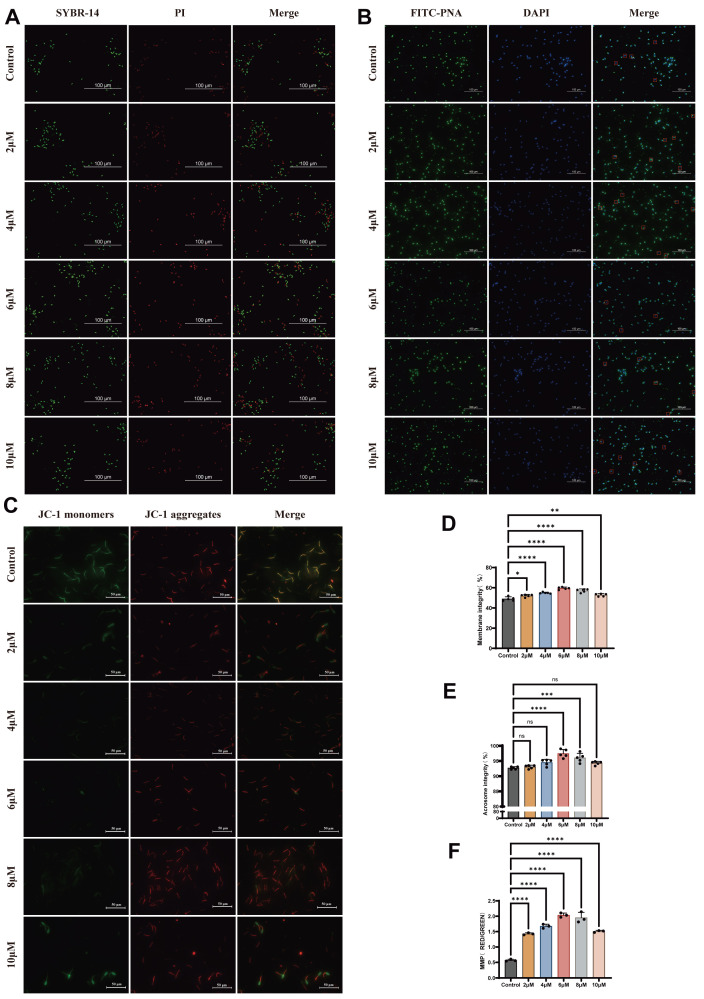
Effects of L-SeMet supplementation on plasma membrane integrity, acrosome integrity, and mitochondrial membrane potential (MMP) of frozen–thawed spermatozoa. (**A**) Representative fluorescence images of sperm plasma membrane integrity evaluated by SYBR-14/PI dual staining. Live spermatozoa exhibited green fluorescence (SYBR-14 positive), whereas dead or membrane-damaged spermatozoa fluoresced red (PI positive). (**B**) Quantitative analysis showing that plasma membrane integrity was significantly higher in the 4, 6, and 8 μM L-SeMet groups than in the control (*p* < 0.05), with the greatest improvement observed at 6 μM. (**C**) Representative fluorescence images of acrosome integrity assessed by FITC-PNA staining (green) and DAPI nuclear counterstaining (blue). Sperm with intact acrosomes displayed uniform green fluorescence, whereas sperm with disrupted acrosomes exhibited weak or absent green signals. Red-framed areas indicate acrosome-damaged spermatozoa. (**D**) Quantitative analysis showing that acrosome integrity was significantly improved in the 6 μM L-SeMet group compared with the control (*p* < 0.05). (**E**) Representative JC-1 fluorescence images showing mitochondrial membrane potential (MMP). High MMP is indicated by red JC-1 aggregates, while low MMP is represented by green monomers. (**F**) Quantitative analysis showing that the 6 μM L-SeMet group exhibited the highest MMP among all groups (*p* < 0.05). Data are presented as mean ± SEM. ns, not significant; * *p* < 0.05; ** *p* < 0.01; *** *p* < 0.001; **** *p* < 0.0001.

To analyze the effects of L-SeMet on post-thawed sperm, we assessed progressive motility (PM) and total motility (TM) using CASA System. Quantitative summaries are presented in Figure 1A–I. The use of extenders following supplementation with 4, 6, 8 μM of L-SeMet resulted in significantly greater sperm PM in comparison to the control group, whereas no significant difference was observed between 2 and 10 μM L-SeMe treatments (*p* < 0.05) (Figure 1A). The addition of 6 µM L-SeMet to the extenders resulted in a significant difference in post-thawed sperm TM compared to the control group (*p* < 0.05) (Figure 1B). Similarly, the VCL and VAP of post-thawed sperm were markedly higher in the group treated with 6 µM L-SeMet compared to the control group (Figure 1D,E). Additionally, other motility parameters did not significantly differ between the L-SeMet-treated groups and the control group (*p* > 0.05) (Figure 1C–I). Together, these data define a narrow optimal window centered at 6 μM, where L-SeMet improves motility and selectively enhances velocity metrics (VCL/VAP), indicating partial preservation of post-thaw sperm quality after cryopreservation. L-SeMet can mitigate the damage membrane integrity, acrosome integrity, and mitochondrial membrane potential (MMP) of sperm. To analyze the impact of adding L-SeMet to the extender I on the plasma membrane integrity rate of the cryopreserved sperm, we assessed the fluorescence intensity in different groups using the SYBR-14/PI double-staining method (Figure 2A). Quantitative data analysis revealed that the plasma membrane integrity of cryopreserved sperm in the experimental groups was significantly enhanced compared to that in the untreated control group, with the differences reaching statistical significance (*p* < 0.05) (Figure 2D). The integrity of the acrosome integrity can be assessed through FITC-PNA staining (Figure 2B). The rate was maximal at a concentration of 6 μM L-SeMet, with statistically significant differences observed compared to control treatment group (*p* < 0.05) (Figure 2E). In contrast, no statistically significant differences were detected between the treatment groups receiving 2, 4, 10 μM L-SeMet and the untreated control group (*p* > 0.05). The JC-1 assay, a widely used method for assessing mitochondrial membrane potential (MMP), was applied to post-thaw sperm treated with six concentrations of L-SeMet (0, 2, 4, 6, 8, and 10 μM). Compared with the normal group, the addition of L-SeMet was found to result in a marked increase inMMP (Figure 2C–F). In conclusion, the results suggest that L-SeMet efficiently mitigates the adverse effects of cryopreservation on sperm membrane integrity, acrosome integrity, and MMP of frozen-thawed spermatozoa. Overall, L-SeMet attenuates cryo-induced damage in post-thaw sperm, affecting the plasma membrane, acrosome, and mitochondria, with maximal protective effects observed at 6 μM. Based on our results demonstrating that supplementation with L-SeMet in the extender mitigated cryo-induced damage and improved post-thaw sperm motility and CASA-derived motion performance in dairy goats, this study aimed to elucidate the metabolic alterations associated with this supplementation. To this end, non-targeted metabolomics analysis was performed to investigate the effect of L-SeMet supplementation during sperm cryopreservation.

### 3.2. Multi-Omics Analyses Reveal the Key Mechanisms by Which L-Selenometionine Protects Spermatozoa

**Figure 3:**
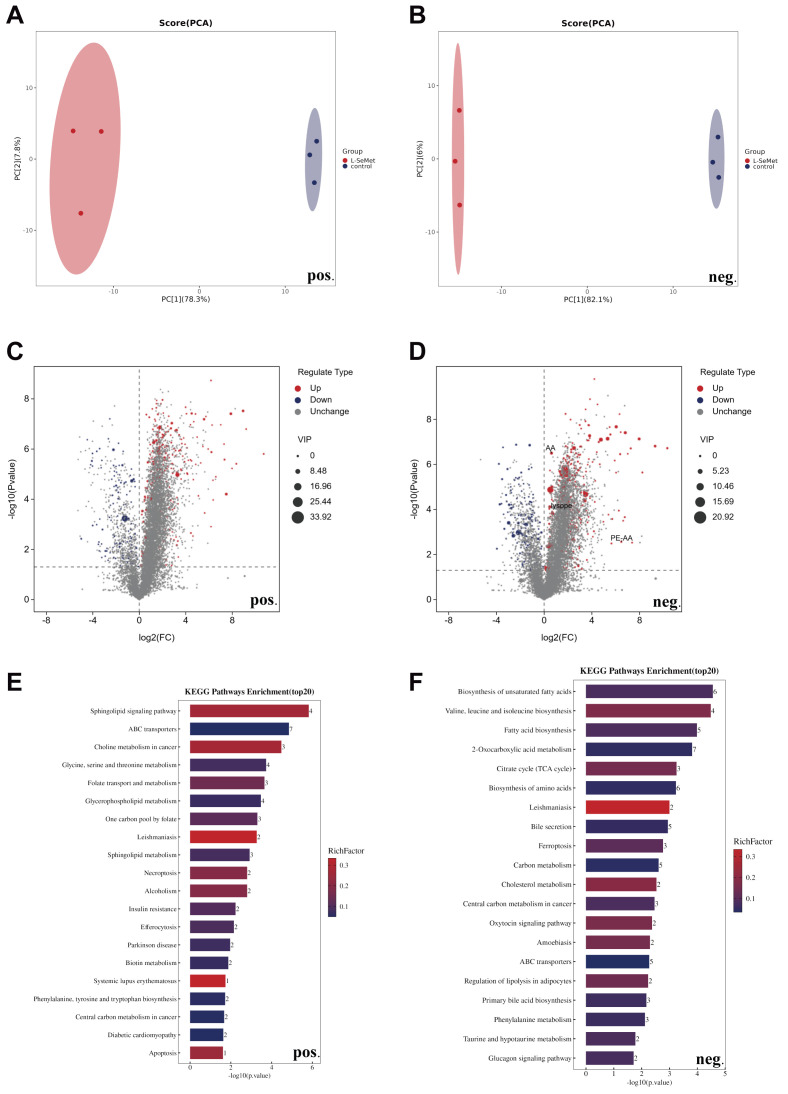
Non-targeted Metabolomic profiling reveals that L-SeMet supplementation remodels metabolic pathways related to lipid, energy, and amino acid metabolism in frozen–thawed spermatozoa. (**A**,**B**) Principal component analysis (PCA) plots in positive-ion (**A**) and negative-ion (**B**) modes showing distinct clustering between the control and L-SeMet-treated groups, indicating substantial metabolic differentiation after supplementation. (**D**) Volcano plots displaying the distribution of significantly altered metabolites in positive (**C**) and negative (**D**) ion modes. Red and blue dots represent upregulated and downregulated metabolites, respectively (VIP > 1, *p* < 0.05). In the negative-ion mode (**D**), arachidonic acid (AA) and arachidonoyl-phosphatidylethanolamine (PE-AA), recognized substrates of lipid peroxidation in ferroptosis, were among the major upregulated metabolites, suggesting enhanced phospholipid remodeling. (**F**) KEGG pathway enrichment analysis of significantly altered metabolites under positive (**E**) and negative (**F**) ion detection modes. In the positive mode, enriched pathways included sphingolipid signaling, ABC transporters, folate metabolism, and glycine/serine/threonine metabolism. In the negative mode, pathways associated with unsaturated fatty acid biosynthesis, the tricarboxylic acid (TCA) cycle, amino acid metabolism, and ferroptosis were significantly enriched. These findings indicate that L-SeMet supplementation modulates lipid and energy metabolism, contributing to redox stabilization during sperm cryopreservation.

Principal component analysis (PCA) revealed clear separation between the control and L-SeMet groups under both positive and negative ion modes, indicating marked differences in their overall metabolic profiles (Figure 3A,B). In the positive ion mode, volcano plots identified a large number of significantly altered metabolites between the two groups, with several metabolites upregulated following L-SeMet treatment (Figure 3C). KEGG enrichment analysis showed that these metabolites were mainly involved in sphingolipid signaling, ABC transporters, folate metabolism, glycine/serine/threonine metabolism, and apoptosis-related pathways (Figure 3E). These pathways are closely associated with processes such as membrane structure, metabolite transport, one-carbon metabolism, amino acid utilization, and programmed cell death. In the negative ion mode, volcano plot analysis identified a total of 94 significantly altered metabolites between the two groups, of which 82 were upregulated and 12 were downregulated following L-SeMet treatment (Figure 3D). KEGG enrichment analysis revealed that these metabolites were significantly enriched in pathways related to unsaturated fatty acid biosynthesis, the tricarboxylic acid (TCA) cycle, amino acid metabolism, and regulation of lipolysis (Figure 3F). These pathways reflect changes in lipid metabolism, central energy metabolism, and amino acid turnover during cryopreservation.

Together, these results demonstrate that L-SeMet supplementation leads to substantial alterations in the sperm metabolome under both positive and negative ion detection modes, with distinct sets of enriched metabolic pathways identified in each mode.

**Figure 4:**
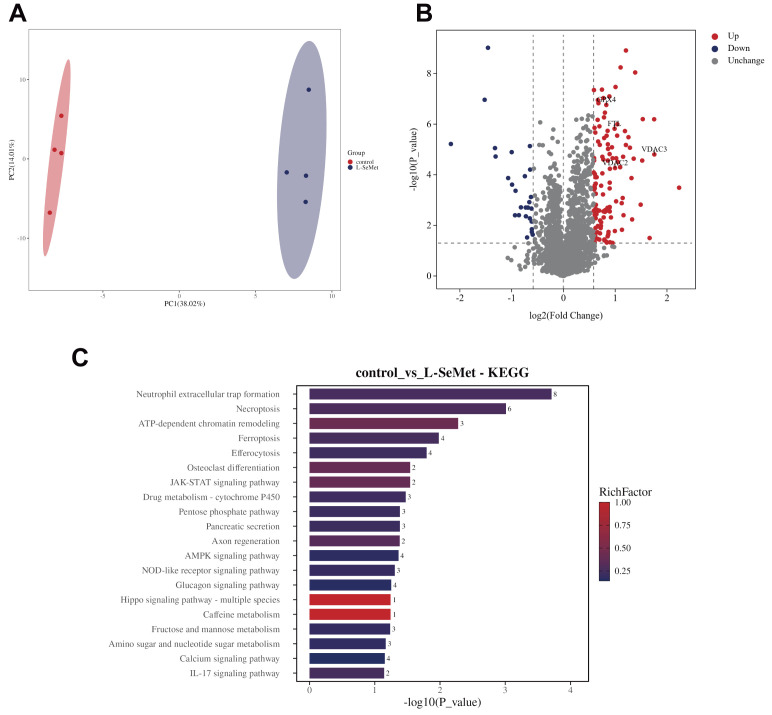
Proteomic profiling of frozen–thawed spermatozoa following L-selenomethionine (L-SeMet) supplementation. (**A**) Principal component analysis (PCA) showing distinct clustering between the control and L-SeMet groups, indicating clear separation of global protein expression profiles. (**B**) Volcano plot displaying significantly differentially expressed proteins between the two groups (|log_2_ fold change| > 1, *p* < 0.05). Proteins related to mitochondrial function and redox regulation, including GPX4, FTL, VDAC2, and VDAC3, were among the significantly upregulated targets. (**C**) KEGG pathway enrichment analysis of differentially expressed proteins. Enriched pathways included ferroptosis, necroptosis, AMPK signaling, and JAK–STAT signaling. A total of 148 proteins (121 upregulated, 27 downregulated) were identified as significantly altered in response to L-SeMet supplementation.

DIA-based quantitative proteomics revealed distinct protein expression profiles between the control and L-SeMet groups. PCA demonstrated clear separation of the two groups, indicating that the improved survival seen in the L-SeMet group was not due to selection bias (Figure 4A). Compared with the control group, a total of 148 proteins were found to be significantly altered in abundance in the L-SeMet group, of which 121 were upregulated and 27 were downregulated, including GPX4, FTH1, VDAC2, and VCDA3. This broad distribution of protein changes highlights the extensive impact of L-SeMet supplementation on the proteome during cryopreservation (Figure 4B). We were surprised that ferroptosis-related proteins were markedly enriched in the KEGG analysis, which contrasts with earlier studies that emphasized apoptosis as the main mechanism of sperm cryoinjury [22,23]. This probably explains that the addition of L-SeMet provides protection by specifically mitigating ferroptosis, a form of regulated cell death characterized by lipid peroxidation and iron overload, rather than by simply inhibiting apoptosis. The enrichment of other cell death pathways, including necroptosis and efferocytosis, further suggests that multiple regulated cell death processes had occurred during cryopreservation (Figure 4C).

Because L-SeMet is a selenium-containing amino acid with potent antioxidant properties, its supplementation may stabilize mitochondrial function and membrane integrity by reducing lipid peroxidation, thereby suppressing ferroptosis. These results showed that protein alterations in the L-SeMet group were closely associated with ferroptosis regulation, highlighting ferroptosis as a critical target in cryodamage and underscoring the importance of L-SeMet in preserving sperm motility during cryopreservation. Collectively, proteome-wide changes induced by L-SeMet supplementation—particularly the up-regulation of GPX4, FTL, VDAC2, and VDAC3, along with the enrichment of ferroptosis-related and stress-response pathways—indicate extensive remodeling of proteins involved in lipid-peroxidation control, iron homeostasis, and mitochondrial function during sperm cryopreservation.

### 3.3. L-SeMet Protects Dairy Goat Spermatozoa from Cryo-Injury by Maintaining Mitochondrial Integrity and Activating the NRF2–SLC7A11–GPX4 Axis

#### 3.3.1. TEM Reveals That L-SeMet Preserves Mitochondrial Ultrastructure During Cryopreservation

**Figure 5:**
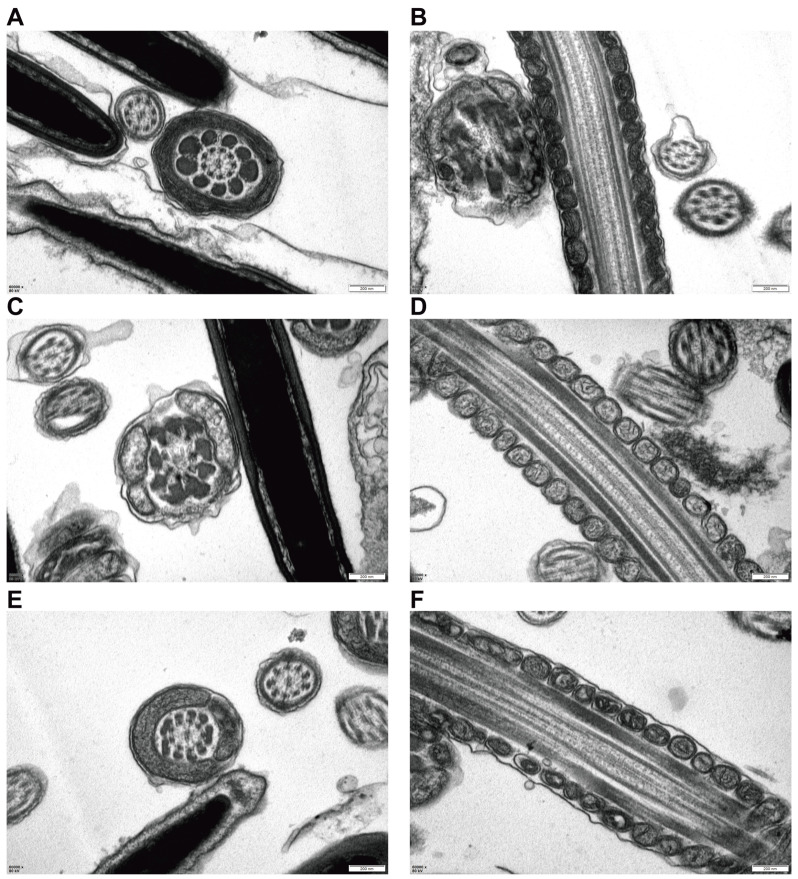
Transmission electron microscopy (TEM) analysis of mitochondrial ultrastructure in frozen–thawed spermatozoa following L-selenomethionine (L-SeMet) supplementation. (**A**,**B**) Representative TEM image of fresh spermatozoa showing normal midpiece ultrastructure with intact double mitochondrial membranes and well-organized cristae. (**C**,**D**) Cryopreserved control spermatozoa displaying severe mitochondrial damage, including disrupted outer membranes, swollen mitochondria, and disorganized or lost cristae. (**E**,**F**) Spermatozoa treated with 6 μM L-SeMet showing improved mitochondrial morphology, characterized by preserved double membranes, dense matrix, and clearly defined cristae. Scale bars = 200 nm.

Transmission electron microscopy (TEM) provided direct morphological evidence of cryo-induced mitochondrial damage in sperm (Figure 5). In fresh samples, mitochondria displayed intact double membranes with well-organized cristae, consistent with normal functional morphology. In contrast, sperm from the cryopreserved control group exhibited marked structural disruptions, including mitochondrial swelling, cristae disintegration, and vacuolization, features that are indicative of severe mitochondrial injury. Notably, supplementation with L-SeMet markedly alleviated these alterations, as mitochondria retained relatively preserved membranes and discernible cristae compared with the control group. These ultrastructural observations align with the proteomic and metabolomic findings, in which ferroptosis-related pathways were significantly enriched. Given that ferroptosis is closely linked to iron-dependent lipid peroxidation and mitochondrial dysfunction, the TEM results support the interpretation that L-SeMet exerts its protective effect by stabilizing mitochondrial integrity and thereby mitigating ferroptosis during cryopreservation.

#### 3.3.2. L-SeMet Improves Post-Thaw Sperm Quality in Dairy Goats by Suppressing Cryo-Induced Ferroptosis Through the NRF2–SLC7A11–GPX4 Signaling Axis

**Figure 6:**
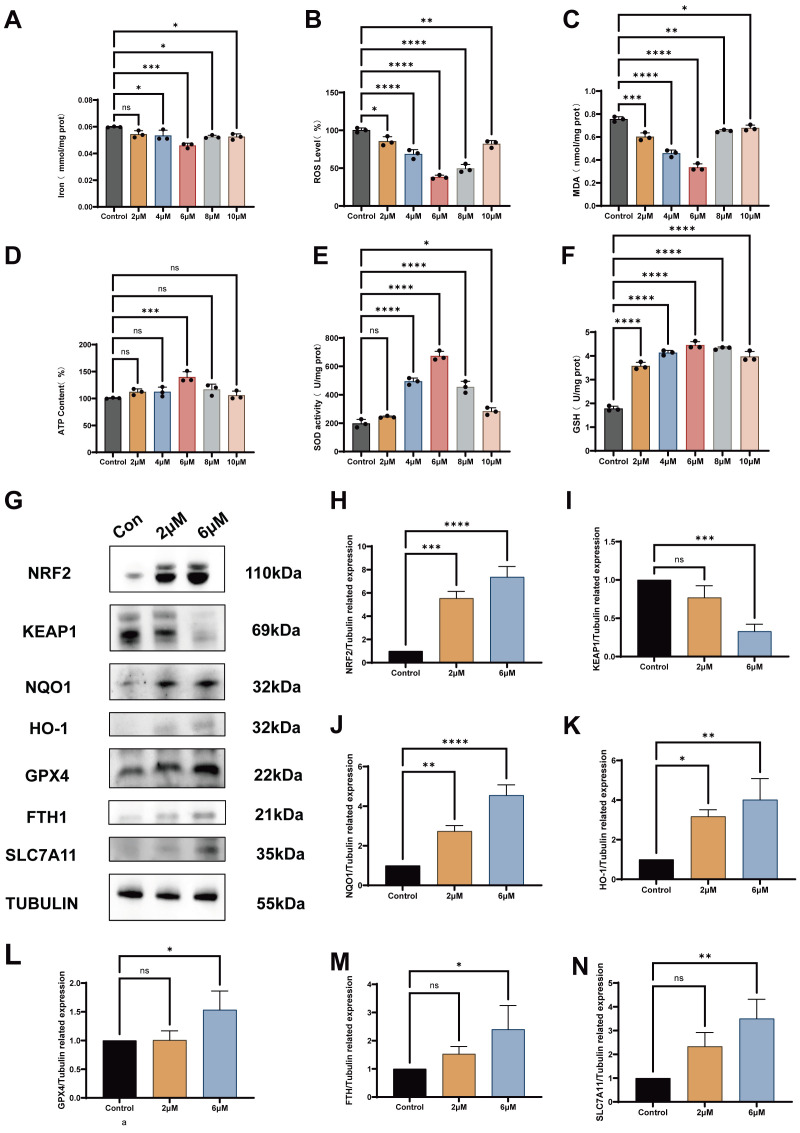
Effects of different concentrations of L-selenomethionine (L-SeMet) on oxidative/antioxidant balance and ferroptosis-related protein expression in frozen–thawed spermatozoa. (**A**–**F**) Biochemical indices of oxidative stress and antioxidant capacity in post-thaw sperm supplemented with varying concentrations of L-SeMet (0, 2, 4, 6, 8, and 10 μM). (**A**) Intracellular iron content, (**B**) reactive oxygen species (ROS), and (**C**) malondialdehyde (MDA) levels were significantly reduced in the 4, 6, 8, and 10 μM groups compared with the control (*p* < 0.05). (**D**) ATP levels were significantly elevated in the 6 μM group (*p* < 0.05). (**E**) Superoxide dismutase (SOD) activity increased significantly at 4, 6, 8, and 10 μM (*p* < 0.05), and (**F**) glutathione (GSH) concentrations were higher in all L-SeMet-treated groups (2–10 μM) than in the control (*p* < 0.05). (**G**) Representative Western blot images of ferroptosis-related proteins, including NRF2, NQO1, HO-1, GPX4, FTH1, SLC7A11, and KEAP1, with TUBULIN as the internal control. (**H**–**N**) Quantitative analysis of relative protein expression levels. Supplementation with 6 μM L-SeMet significantly increased NRF2, NQO1, HO-1, GPX4, FTH1, and SLC7A11 expression (*p* < 0.05), while KEAP1 expression was markedly decreased (*p* < 0.05). Data are presented as mean ± SEM. ns, not significant; * *p* < 0.05; ** *p* < 0.01; *** *p* < 0.001; **** *p* < 0.0001.

Biochemical indices were quantified in post-thaw sperm across L-SeMet doses (0, 2, 4, 6, 8, 10 μM), followed by immunoblotting at 6 μM (Figure 6). We found that dose-dependent changes in multiple oxidative and antioxidant parameters in post-thaw sperm following L-SeMet supplementation (Figure 6). Iron content was significantly reduced in the 4, 6, 8, and 10 μM groups compared with the control (*p* < 0.05; Figure 6A). ROS levels were also significantly decreased at 4, 6, 8, and 10 μM relative to control (*p* < 0.05; Figure 6B). In addition, MDA concentrations were consistently lower across all L-SeMet-treated groups (2–10 μM) than in the control (*p* < 0.05; Figure 6C). By contrast, ATP levels showed a significant increase only in the 6 μM group (*p* < 0.05; Figure 6D). Moreover, SOD activity was significantly elevated at 4, 6, 8, and 10 μM compared with the control (*p* < 0.05; Figure 6E), while GSH concentrations were significantly increased in all L-SeMet-treated groups (2–10 μM) (*p* < 0.05; Figure 6F). Western blot analysis was performed to confirm the effects of L-SeMet on ferroptosis-related proteins in post-thaw sperm (Figure 6G–M). Compared with the cryo control, supplementation with 6 μM L-SeMet resulted in a significant increase in the protein abundance of NRF2, NQO1, HO-1, GPX4, FTH1, and SLC7A11 (all *p* < 0.05). By contrast, the expression of KEAP1 was significantly decreased (*p* < 0.05). All results were normalized to TUBULIN as the internal loading control. These findings indicate that 6 μM L-SeMet consistently promoted the activation of antioxidant and ferroptosis-regulatory proteins while suppressing KEAP1, aligning with the biochemical improvements observed in iron, ROS, MDA, ATP, SOD, and GSH levels.

**Figure 7:**
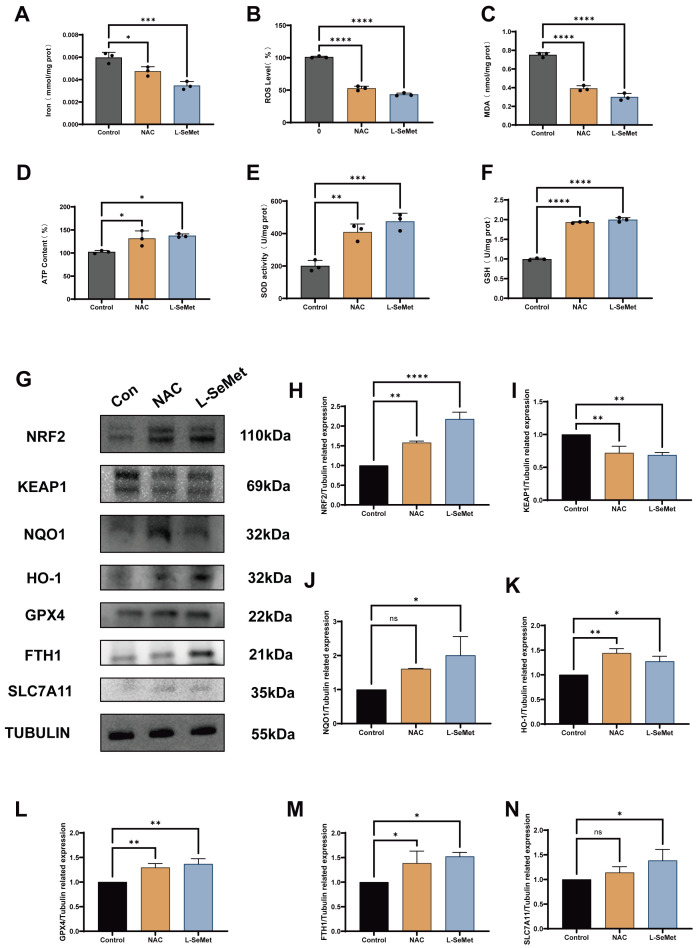
Effects of the ferroptosis inhibitor NAC and L-selenometionine (L-SeMet) on oxidative/antioxidant indices and ferroptosis-related proteins in frozen–thawed spermatozoa. (**A**–**F**) Biochemical measurements in post-thaw sperm from control, NAC, and L-SeMet groups: (**A**) intracellular iron, (**B**) reactive oxygen species (ROS), (**C**) malondialdehyde (MDA), (**D**) ATP, (**E**) superoxide dismutase (SOD) activity, and (**F**) glutathione (GSH). Both NAC and L-SeMet significantly reduced iron, ROS, and MDA and increased ATP, SOD, and GSH compared with control (*p* < 0.05). (**G**) Representative immunoblots of NRF2, NQO1, HO-1, GPX4, FTH1, SLC7A11, and KEAP1; TUBULIN served as the loading control. (**H**–**N**) Densitometric quantification of the proteins shown in (**G**). NAC and L-SeMet groups exhibited higher levels of NRF2 (**H**), NQO1 (**I**), HO-1 (**J**), GPX4 (**K**), FTH1 (**L**), and SLC7A11 (**M**) and lower KEAP1 (**N**) relative to control (*p* < 0.05). No significant differences were detected between NAC and L-SeMet for these indices (*p* > 0.05). Data are presented as mean ± SEM. ns, not significant; * *p* < 0.05; ** *p* < 0.01; *** *p* < 0.001; **** *p* < 0.0001.

To further validate whether the protective effects of L-SeMet were related to ferroptosis inhibition, we compared post-thaw sperm treated with the ferroptosis inhibitor NAC to those supplemented with L-SeMet (Figure 7). Biochemical assays were conducted to evaluate the effects of NAC and L-SeMet on post-thaw sperm (Figure 7A–F). Both treatments significantly reduced iron content, ROS levels, and MDA concentrations compared with the cryo control group (all *p* < 0.05). In contrast, ATP content, SOD activity, and GSH concentrations were significantly higher in both NAC- and L-SeMet-treated groups than in the control (all *p* < 0.05). No significant differences were observed between NAC and L-SeMet groups for any of these indices. Western blot analysis further confirmed these findings (Figure 7G–M). Compared with controls, both NAC and L-SeMet treatment resulted in higher expression of NRF2, NQO1, HO-1, GPX4, FTH1, and SLC7A11, accompanied by a reduction in KEAP1 levels (all *p* < 0.05). The expression patterns were similar between NAC and L-SeMet groups. Together, these results demonstrate that supplementation with L-SeMet exerts protective effects on post-thaw sperm comparable to those of the ferroptosis inhibitor NAC, as reflected by improvements in oxidative/antioxidant balance and consistent modulation of NRF2–KEAP1 pathway proteins.

**Figure 8:**
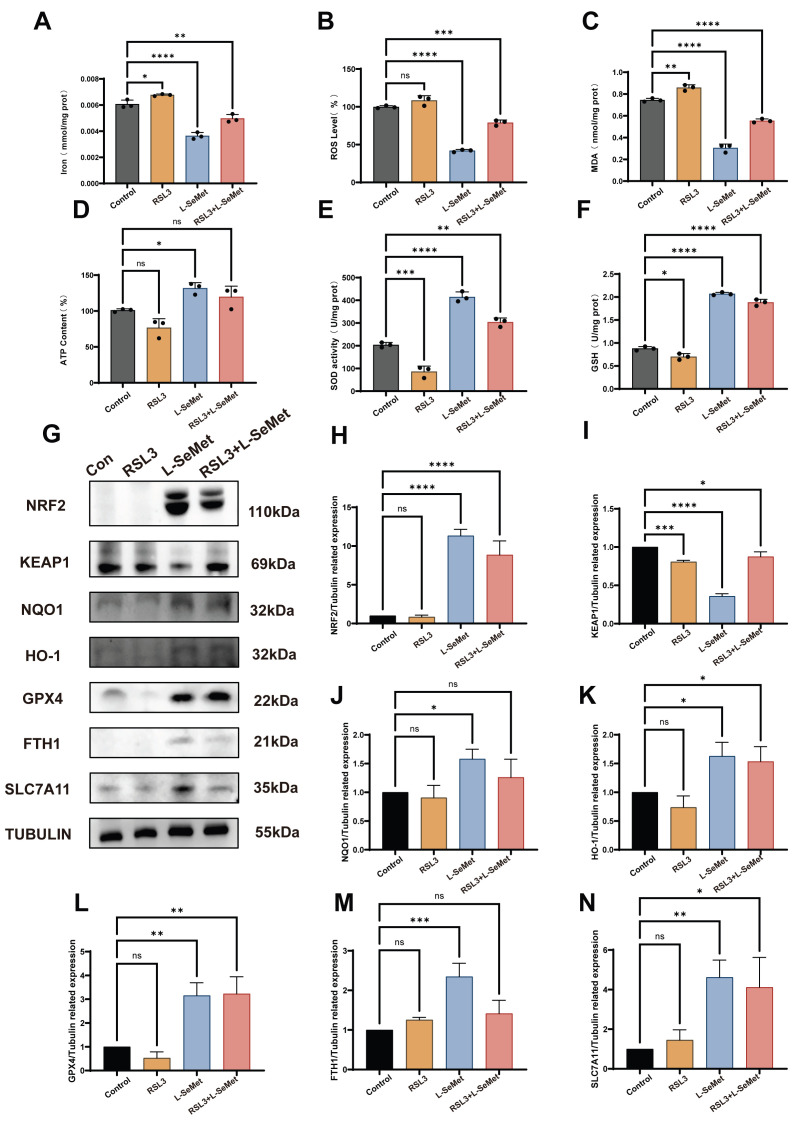
Effects of the ferroptosis inducer RSL3 and L-selenomethionine (L-SeMet) co-incubation on oxidative/antioxidant indices and ferroptosis-related protein expression in frozen–thawed spermatozoa. (**A**–**F**) Biochemical measurements in post-thaw sperm from control, RSL3 (5 μM), L-SeMet (6 μM) groups and RSL3+ L-SeMet (6 μM) groups: (**A**) intracellular iron content, (**B**) reactive oxygen species (ROS), (**C**) malondialdehyde (MDA), (**D**) ATP, (**E**) superoxide dismutase (SOD) activity, and (**F**) glutathione (GSH). Compared with the control, RSL3 significantly increased iron, ROS, and MDA while decreasing ATP, SOD, and GSH (*p* < 0.05). Co-incubation with L-SeMet effectively reversed these alterations (*p* < 0.05). (**G**) Representative Western blot images of ferroptosis-related proteins, including NRF2, KEAP1, NQO1, HO-1, GPX4, FTH1, and SLC7A11; TUBULIN was used as a loading control. (**H**–**N**) Quantitative analysis of protein expression levels. RSL3 exposure suppressed NRF2, NQO1, HO-1, and GPX4 (**H**–**K**) while upregulating KEAP1 (**I**) and inducing compensatory increases in FTH1 (**M**) and SLC7A11 (**N**) compared with the control (*p* < 0.05). L-SeMet co-treatment significantly restored NRF2–NQO1–HO-1–GPX4 axis activation, reduced KEAP1 levels, and moderated the overexpression of FTH1 and SLC7A11 induced by iron overload (*p* < 0.05). Data are presented as mean ± SEM. ns, not significant; * *p* < 0.05; ** *p* < 0.01; *** *p* < 0.001; **** *p* < 0.0001.

To further examine whether L-SeMet attenuates ferroptosis, post-thaw sperm were exposed to the ferroptosis inducer RSL3 (5 μM), with or without co-incubation of 6 μM L-SeMet (Figure 8). Compared with the cryo control, RSL3 treatment significantly increased iron content, ROS levels, and MDA concentrations, while significantly reducing ATP content, SOD activity, and GSH concentrations (all *p* < 0.05; Figure 8A–F). Co-incubation with L-SeMet partially reversed these alterations: iron, ROS, and MDA levels were significantly decreased, and ATP, SOD, and GSH were significantly increased compared with the RSL3 group (all *p* < 0.05). Western blot analysis further demonstrated distinct protein expression changes (Figure 8G–M). In sperm treated with RSL3, the expression of NRF2, NQO1, HO-1, and GPX4 was suppressed, whereas FTH1 and SLC7A11 were elevated, and KEAP1 was upregulated compared with the control (all *p* < 0.05). Importantly, co-incubation with L-SeMet significantly restored the expression of NRF2, NQO1, HO-1, and GPX4, reduced KEAP1, and moderated the compensatory elevations of FTH1 and SLC7A11 induced by RSL3 (all *p* < 0.05).

## 4. Discussion

Cryopreservation rapidly disrupts redox homeostasis and compromises membrane integrity in spermatozoa, resulting in mitochondrial dysfunction and reduced motility. L-SeMet, the predominant organic form of selenium in natural diets, exhibits higher bioavailability, lower toxicity, and greater efficiency of protein incorporation than inorganic selenium sources such as sodium selenite or selenate [24,25,26,27]. Within cells, L-SeMet can be nonspecifically incorporated into proteins in place of methionine or converted to selenocysteine through trans-selenation, thereby directly supporting selenoprotein synthesis [28,29,30]. Among selenoproteins, glutathione peroxidase 4 (GPX4) functions as a key antioxidant enzyme that detoxifies lipid hydroperoxides within biological membranes and acts as a molecular gatekeeper against ferroptosis [31,32,33]. Given its dual role as a selenium donor and as an enhancer of cellular antioxidant defense, L-SeMet is expected to counteract cryo-induced ferroptotic damage in spermatozoa. Here, we demonstrate that supplementation with L-SeMet during sperm cryopreservation protects post-thaw sperm by attenuating ferroptosis, with the maximal protective effect observed at 6 μM.

Our CASA analysis shows that L-SeMet supplementation improved post-thaw motility at 4–8 μM, with a maximal effect at 6 μM, and selectively increased motility, VCL, and VAP at 6 μM, whereas other kinematic readouts remained unchanged. This phenomenon may be attributed to the dose-dependent dual actions of selenium, which exerts antioxidant and cytoprotective effects at low concentrations but becomes pro-oxidative and potentially cytotoxic at higher levels. For instance, Choi et al. reported that high-dose selenium in ovarian cancer cells elevated ROS levels, suppressed GPX4 expression, and triggered lipid peroxidation, consistent with ferroptotic cell death [34]. Moreover, the concept of reductive stress—an imbalance caused by excessive reducing equivalents shifting redox homeostasis toward a hyper-reduced state—has also been proposed as a mechanism of high-dose selenium toxicity [35]. In buffalo and bovine models, adding low-μg/mL selenium to extenders improved frozen–thawed motility, often with corroboration from CASA kinematics and membrane-integrity readouts, whereas deviations from the optimal range did not confer additional benefit [8,36]. Likewise, nano-selenium around ~1 μg/mL elevated motility in rams [37].

In our study, the absence of improvement at 10 μM likely reflects disruption of redox balance or the onset of reductive stress-associated side effects beyond the optimal protective range. This dose-screening experiment served to determine an effective working concentration of L-SeMet for our cryopreservation protocol. A plausible biological explanation is that L-SeMet partially restores post-thaw energetic capacity and membrane functionality, rather than broadly altering sperm track linearity or flagellar beat patterns. Given that VCL and VAP are tightly coupled to ATP availability and flagellar power output, the pronounced improvement observed at 6 μM indicates enhanced mitochondrial support for axonemal activity within this concentration range [38,39]. Collectively, these findings indicate that 6 μM L-SeMet is an optimal working concentration for downstream analyses in this cryopreservation model.

Subsequently, we analyzed the same endpoint across distinct sperm compartments, including plasma membrane integrity (SYBR-14/PI), acrosomal status (FITC-PNA), and mitochondrial function (JC-1), thereby providing complementary evidence for the protective effects of L-SeMet during cryopreservation. From a biological perspective, the pattern is functionally coherent. Plasma-membrane integrity generally responds across a broad range of antioxidant exposures, whereas acrosomal structure and mitochondrial potential serve as more sensitive discriminators of cryo-injury [40,41]. Using the same thawing protocol, L-SeMet increased the proportion of membrane-intact sperm at all concentrations, indicating broad tolerance and intrinsic protective effects. Acrosomal integrity and mitochondrial membrane potential both peaked at 6 μM, whereas other doses produced smaller or inconsistent effects. When viewed in the context of contemporary selenium literature, our findings are consistent with dose-dependent responses across species. In bull semen, supplementation with Se nanoparticles (with or without glutathione) at moderate concentrations improved plasma membrane integrity, acrosomal integrity, and mitochondrial membrane potential during cryopreservation [42]. In boar semen preserved in liquid form, approximately 1 µg/mL SeNPs produced the highest semen quality and conception rates [43]. By contrast, studies in ram spermatozoa using excess inorganic selenium or high-dose SeNPs have reported reduced post-thaw motility and compromised membrane integrity [36]. Collectively, this body of evidence supports a biphasic dose–response pattern, in which intermediate L-SeMet levels confer concordant improvements in structural (acrosomal, membrane) and energetic (mitochondrial) parameters—mirroring the convergence observed at 6 μM in our study. Notably, this convergence parallels the motility enhancements detected at the same concentration, reinforcing the functional coherence of the protective response.

Supplementation with L-SeMet during cryopreservation markedly improved post-thaw sperm quality by enhancing motility, and membrane integrity. To elucidate the underlying protective mechanism of L-SeMet, we employed an integrated DIA-based quantitative proteomic and untargeted metabolomic approach. Both datasets revealed distinct separation between L-SeMet-treated and control sperm, and KEGG enrichment analysis showed that differentially expressed proteins and metabolites were significantly associated with ferroptosis-related pathways. In negative-ion mode, our metabolomics highlighted arachidonic acid (AA) and arachidonoyl-phosphatidylethanolamine (PE-AA) species—canonical substrates for ferroptotic lipid peroxidation—together with pathway signals consistent with PUFA–phospholipid (PUFA-PL) remodeling. This profile maps directly onto the biochemical core of ferroptosis: ACSL4 activates AA to AA-CoA and, with LPCAT3, incorporates it into PE to generate PE-AA, which is subsequently oxygenated to PE-AA-OOH; GPX4 then reduces these hydroperoxides to their corresponding alcohols, aborting the chain reaction [44].

The enrichment of AA and PE-AA in the L-SeMet group does not contradict the suppression of ferroptosis. Instead, it likely reflects an accumulation of non-oxidized polyunsaturated phospholipids under enhanced antioxidant protection. Kagan et al. [32] showed that under ferroptosis-inhibitory conditions, non-oxidized PUFA-PE species may accumulate significantly, owing to suppressed lipoxygenase activity and bolstered antioxidant defenses. Together, these findings support the interpretation that the observed AA and PE-AA enrichment represents a protective metabolic state in which lipid substrates are preserved rather than oxidized [33]. Rather, in a scenario where iron-catalyzed peroxidation flux is restrained by NRF2 activation, upregulated SLC7A11/GPX4, and ferritin-mediated iron sequestration, a larger steady-state pool of non-oxidized PE-AA can accumulate. Supporting this view, NRF2 is known to regulate genes controlling both lipid and iron homeostasis in ferroptosis contexts [45]. In such a low-flux state, PUFA–PLs are preserved and recycled via acyl-remodeling and membrane-repair, sustaining mitochondrial function without fueling lipid peroxidation [46]. Consistent with this interpretation, our DIA-based proteomics provided direct evidence for these shifts, revealing upregulation of several ferroptosis regulators in the L-SeMet group—most notably GPX4, FTH1, and the mitochondrial porins VDAC2 and VDAC3. Functionally, GPX4 is the only enzyme capable of directly reducing phospholipid hydroperoxides within membranes using glutathione (GSH) as a cofactor, thereby increasing the threshold for lipid-peroxidation chain reactions that define ferroptosis [47]. Selenium supplied by L-SeMet plausibly supports both the abundance and catalytic activity of GPX4 in line with the observed preservation of membrane structure and mitochondrial integrity [48]. Upregulation of ferritin represents the complementary arm of iron homeostasis. Accordingly, the metabolomic accumulation of AA/PE-AA, together with the proteomic upregulation of GPX4, reflects a scenario in which substrates are preserved while enzymatic and iron-handling systems prevent their conversion into cytotoxic lipid hydroperoxides [49].

Ferritin, a 24-subunit nanocage composed of heavy (FTH1) and light (FTL) chains, stores up to ~4500 Fe atoms in a mineralized core [47]. The heavy chain provides ferroxidase activity that converts Fe^2+^ to Fe^3+^ for safe sequestration, while the light chain stabilizes the core structure. This storage sharply reduces the labile Fe^2+^ pool that drives Fenton chemistry and lipid radical propagation. Accordingly, elevated FTH1/FTL limits the initiating flux for ferroptosis, whereas ferritin degradation through NCOA4-mediated ferritinophagy releases iron and sensitizes cells to ferroptotic damage [50]. VDAC2 and VDAC3 are voltage-dependent anion channels located on the outer mitochondrial membrane, responsible for mediating metabolite and ion exchange, and thereby regulating MMP and ATP production.

Previous studies have shown that VDAC3 deficiency in mice leads to damage in the sperm tail structure and disintegration of the flagellum, which contribute to a significant loss of motility and infertility. Furthermore, VDAC2/3 have been implicated in maintaining the structural integrity of the sperm mitochondrial sheath [51,52,53]. Consistent with these findings, we observed that VDAC2/3 upregulation in the L-SeMet-treated group correlated with improved MMP and ATP levels, suggesting that the upregulation of these channels supports mitochondrial stability and energy production during cryopreservation [54]. Despite VDAC2/3 being considered key targets of erastin in inducing ferroptosis, the lack of SLC7A11 upregulation and the reduction in MDA levels in our study argue against ferroptosis induction in this context [55]. Instead, In the context of an enhanced antioxidant defense, the upregulation of VDAC2/3 is more likely to reflect the stabilization of mitochondrial flux and energetic homeostasis rather than a pro-oxidative response.

TEM was conducted to provide ultrastructural validation of the molecular findings, focusing on mitochondrial morphology [56]. In the control group, mitochondria exhibited severe structural damage, including disrupted cristae and membrane fragmentation, further supporting the proteomic, metabolomic, and MMP evidence of ferroptosis activation during cryopreservation [57,58,59]. These findings are consistent with the conclusions of Erhan Hai et al., suggesting that ferroptosis plays a key role in sperm injury [60]. Notably, these ultrastructural lesions were partially reversed by L-SeMet supplementation, highlighting its protective effect against cryo-induced damage.

During cryopreservation and thawing, spermatozoa are exposed to oxidative stress that can impair their physiological function. Many studies indicate that freeze–thawing leads to a significant increase in reactive oxygen species (ROS) levels, which damage spermatozoa and reduce their motility [2]. In parallel, reductions in antioxidant enzyme activity—especially SOD and elevated MDA levels have been frequently reported in post-thaw sperm, consistent with enhanced lipid peroxidation and oxidative injury [61]. Our results align with these findings: cryo-thawing appears to diminish the sperm’s capacity to neutralize ROS, leading to excessive free radical production, depleted antioxidative defenses, and subsequent oxidative damage [2]. By contrast, supplementation with L-Se-Met markedly reduced lipid peroxide levels compared to the untreated control group, thereby preserving sperm motility. To elucidate how L-SeMet modulates ferroptosis-related pathways during sperm cryopreservation, we examined the expression of key proteins governing iron handling and antioxidant defense. Previous studies have established that ferroptosis is driven by enhanced TFRC expression, a downstream target that promotes cellular iron uptake and accumulation [62]. In contrast, overexpression of FTH1, the ferritin heavy chain responsible for iron sequestration, confers resistance to ferroptosis by limiting labile Fe^2+^ and Fenton chemistry [63]. In our study, L-SeMet supplementation restored FTH1 abundance relative to the control, suggesting that selenium normalizes intracellular iron homeostasis and reduces free-iron–driven oxidative burden. This interpretation is supported by the decreased total iron content observed in the biochemical assays, which together with the proteomic data points to coordinated re-establishment of iron storage capacity under L-SeMet treatment.

Moreover, GPX4 is a central ferroptosis suppressor that reduces lipid peroxides using selenium as a cofactor, and selenium utilization by GPX4 is essential to preventing ferroptosis induced by hydroperoxides [48]. SLC7A11, the light chain subunit of the cystine/glutamate antiporter (system Xc^−^), imports cystine into cells for glutathione (GSH) synthesis; its overexpression has been shown to suppress ROS-initiated ferroptosis in tumor cells. In concert, NRF2 is a master regulator of redox homeostasis and can transcriptionally upregulate SLC7A11 and GPX4, thereby preventing ferroptosis in chronic disease contexts [64]. In our experiment, L-SeMet supplementation also increased SLC7A11 expression, which argues against a scenario in which VDAC2/3 prematurely bind erastin and suppress SLC7A11. Instead, this result supports our earlier conjecture that VDAC2/3 in this context are not functioning as erastin-binding “pro-death” channels but rather as components of a coordinated antioxidant response.

Mechanistically, both GPX4 and SLC7A11 have been validated as transcriptional targets of NRF2, placing them under the control of the KEAP1–NRF2 axis [45]. Under basal conditions, Keap1 targets NRF2 for ubiquitin-mediated degradation, whereas upon oxidative stress, NRF2 escapes Keap1 repression, translocates to the nucleus, and activates antioxidant response element (ARE) genes including NQO1 and HO-1. Consequently, the coordinated restoration of FTH1, GPX4, SLC7A11, and modulation of Keap1/NQO1/HO-1 under L-SeMet treatment strongly suggests that selenium reactivates the NRF2–SLC7A11–GPX4 axis, thereby restoring iron redox homeostasis and suppressing ferroptosis [65].

The inclusion of NAC and RSL3 treatment arms was designed to distinguish whether L-SeMet protects sperm through specific inhibition of ferroptosis rather than nonspecific antioxidation. NAC, acting as a cysteine donor and supporting GSH synthesis, reinforces the SLC7A11–GSH–GPX4 axis and thereby limits lipid peroxidation—a mechanism characteristic of classical ferroptosis inhibitors [3,33]. Conversely, RSL3 directly inhibits the enzymatic activity of GPX4, leading to the accumulation of lipid hydroperoxides and subsequent iron-dependent cell death, thereby initiating ferroptosis [66,67]. Exposure of round spermatids to 10 μM RSL3 induced a stage-dependent ferroptotic response, reducing cell viability by approximately 40% within 1 h and confirming that RSL3 rapidly triggers ferroptosis in spermatogenic cells [68,69]. In the validation assays, L-SeMet supplementation produced effects analogous to those of NAC. Notably, co-treatment with L-SeMet partially reversed the deleterious effects of RSL3, restoring redox balance and mitochondrial integrity. These outcomes confirm that the protective action of L-SeMet operates primarily through ferroptosis inhibition rather than general antioxidation [3,7].

## 5. Conclusions

L-SeMet improves post-thaw sperm motility and preserves mitochondrial-driven kinematics within a defined therapeutic window. Multi-omics enrichment, coordinated upregulation of NRF2–SLC7A11–GPX4, reduced iron and oxidative burden, and partial resilience to iron loading collectively indicate that ferroptosis mitigation is the primary route through which L-SeMet sustains sperm function after thawing. Taken together, our data provide strong evidence that L-SeMet alleviates cryo-induced sperm damage via ferroptosis attenuation. Notably, the present evidence is derived exclusively from in vitro assays and should be considered preliminary; further in vivo validation.

## Data Availability

The mass spectrometry proteomics data have been deposited to the ProteomeXchange Consortium (https://proteomecentral.proteomexchange.org, accessed on 1 March 2026) via the iProX partner repository [70,71] with the dataset identifier PXD075034. All other data supporting the findings of this study are available within the article; additional information can be provided by the corresponding author upon reasonable request.
